# Media exposure predicts acute stress and probable acute stress disorder during the early COVID-19 outbreak in China

**DOI:** 10.7717/peerj.11407

**Published:** 2021-05-10

**Authors:** Yu Luo, Xiangcai He, Shaofeng Wang, Jinjin Li, Yu Zhang

**Affiliations:** 1 School of Psychology, Guizhou Normal University, Guiyang, Guizhou, China; 2 Chengdu Medical College, Chengdu, Sichuan, China

**Keywords:** COVID-19, Media exposure, Acute stress, Acute stress disorder, Mental health

## Abstract

**Background:**

The COVID-19 has led to unprecedented psychological stress on the general public. However, the associations between media exposure to COVID-19 and acute stress responses have not been explored during the early COVID-19 outbreak in China.

**Methods:**

An online survey was conducted to investigate the relationships between media exposure to COVID-19 and acute stress responses, and to recognize associated predictors of acute stress responses on a sample of 1,450 Chinese citizens from February 3 to February 10, 2020. Media exposure questionnaire related to COVID-19 was developed to assess media exposure time, media exposure forms and media exposure content. The Stanford Acute Stress Reaction Questionnaire (SASRQ) was used to measure acute stress responses, including continuous acute stress symptom scores and the risk of probable acute stress disorder (ASD). A series of regression analyses were conducted.

**Results:**

Longer media exposure time and social media use were associated with higher acute stress and probable ASD. Viewing the situation of infected patients was associated with higher acute stress, whereas viewing the latest news about pandemic data was associated with lower odds of probable ASD. Being females, living in Hubei Province, someone close to them diagnosed with COVID-19, history of mental illness, recent adverse life events and previous collective trauma exposure were risk factors for acute stress responses.

**Conclusions:**

These findings confirmed the associations between indirect media exposure to pandemic events and acute stress responses. The governments should be aware of the negative impacts of disaster-related media exposure and implement appropriate interventions to promote psychological well-being following pandemic events.

## Introduction

In December 2019, a novel coronavirus disease (COVID-19) was first reported in Wuhan, the capital city of Hubei Province, China, and then started to spread throughout China (Chan et al., 2020). The COVID-2019 has spread very rapidly to many other countries around the globe, leading to continuously increasing confirmed cases (Bao et al., 2020). Due to the rapid spread of COVID-19, most provinces in China initiated the Level-1 emergency response plans to cope with the contagious pandemic outbreak by January 25, 2020. Next, the Chinese government imposed the lockdown, the restricted transportation and the home quarantines as emergency measures to prevent the spread of COVID-19. However, with the increasing of confirmed cases, the circumstance was becoming progressively serious. On January 30, 2020, the World Health Organization (WHO) declared that the COVID-19 outbreak was a global public health emergency (World Health Organization, 2020). During the early COVID-19 outbreak, many aspects of life were full of unknowns, and the public had a great need for the latest information about COVID-19 from the media to make sense of the situation and to protect their health (Chao et al., 2020; Gao et al., 2020; Garfin, Silver & Holman, 2020). Generally, media coverage is productive to provide some relief from panic, reduce uncertainty and exacerbate perceptions of personal health following the COVID-19 outbreak. However, long-term and repeated media exposure may extend the boundary of the disaster itself (Vasterman, Yzermans & Dirkzwager, 2005; Thompson et al., 2019), which can cause some negative impacts on media consumers with no direct personal exposure to disaster events.

Previous studies have elucidated that disaster-related media exposure is associated with negative psychological outcomes (Holman, Garfin & Silver, 2014; Yeung et al., 2016; Hall et al., 2019). For example, Silver et al. (2013) found that repeatedly engaging with trauma-related media content could prolong acute stress experiences and promote substantial stress-related symptomatology after the 9/11 terrorist attacks. Meanwhile, some empirical research suggests that disaster-related media exposure is linked to acute stress responses (Goodwin et al., 2015; Holman, Garfin & Silver, 2014; Thompson et al., 2019). For example, accumulated evidence has indicated that frequently engaging with trauma-related media content could increase acute stress symptoms following the Boston Marathon bombings (Holman, Garfin & Silver, 2014; Holman et al., 2019; Thompson et al., 2019). However, most of these studies mainly focused on man-made disasters (e.g., terrorist attack) and natural disasters (e.g., typhoon). Only a few studies examined the links between media exposure and psychological distress during the pandemic outbreak (e.g.,  Thompson et al., 2017). Since the COVID-19 outbreak, several studies have attempted to reveal the associations between media use and mental health (e.g.,  Elhai et al., 2020; Chao et al., 2020; Holman et al., 2020). So far, however, the research about the relationships between media exposure to COVID-19 and acute stress responses during the early COVID-19 outbreak in China is relatively limited.

Acute stress response refers to a series of physiological and psychological reactions, which is usually triggered by a stressful and life-threatening event (Pappas et al., 2009). The COVID-19 pandemic, as a public health event, is featured by its rapid transmission, uncertainty about future, considerable mortality and serious impacts (Ye et al., 2020). The stressful experiences from either the outbreak itself or the subsequent government responses to the outbreak (e.g., lockdown, travel restrictions) occurred in a very short time period following the COVID-19 outbreak, which may lead to the increase of subsequent COVID-19-related acute stress responses. Furthermore, the ongoing perceived threats, inconsistent information and uncertainty about the future, accompanied by the pandemic may contribute to the development of acute stress disorder (ASD) by prolonging or exacerbating acute trauma-related symptoms. As a predictor of post-traumatic stress disorder (PTSD), ASD is one of the early psychological disorders after disasters (Bryant, 2011; Gnanavel & Robert, 2013). If the public have been in a state of ASD for a long time without interventions, they will easily suffer from PTSD shortly afterwards (Bryant, 2005; Fuglsang, Moergeli & Schnyder, 2004; Zhou et al., 2015). Therefore, it is necessary to explore the associations between pandemic-related media exposure and acute stress responses during the early COVID-19 outbreak, and to provide reference data for the psychological prevention and intervention.

### Media exposure time and acute stress responses

Media is found to facilitate the dissemination of latest available information to improve knowledge, awareness, and practices of general public (Gralinski & Menachery, 2020; Houston, Spialek & First, 2018). However, different media exposure time, media exposure forms and media exposure content may have distinct effects on psychological outcomes during the COVID-19 pandemic. Several studies have found that higher average time spent on media after the disaster-related events is associated with more psychological problems (Holman, Garfin & Silver, 2014; Liu & Liu, 2020; Thompson et al., 2019; Yeung et al., 2016). For example, Hossain et al. (2020) found that individuals with COVID-19-related social media exposure of over four hours per day experienced higher levels of anxiety than individuals with less than or equal to 2 h social media exposure to COVID-19. However, less is unknown whether media exposure time is associated with acute stress responses (i.e., acute stress and probable ASD) during the early COVID-19 outbreak in China.

### Media exposure forms and acute stress responses

Different media exposure forms may have distinct associations with acute stress responses. Earlier studies mainly focused on the engagement with traditional media. For example, a study from the United States revealed that television viewing of 9/11-related coverage was associated with the likelihood of PTSD (Ahern et al., 2002). Traditional media, like television, usually appears to provide relatively objective statements about threat (Lemyre, Johnson & Corneil, 2010). However, with the development of media technology, some new media forms including online news (e.g., Toutiao, Xinhuanet) and short video application (e.g., Tik Tok, Kuaishou), have become the important media sources of indirect trauma-related exposure. New media usually has more direct, personal impacts on risk assessments, which can offer more opportunities for the emotional contagion related to the risk appraisal and stress after traumatic events (Mineka & Zinbarg, 2006). Nevertheless, there are no consistent conclusions regarding the effects of different media exposure forms on psychological health (Chao et al., 2020). For example, Rodgers et al. (2012) found that the use of traditional media (e.g., television) was related to more psychological distress among adults compared with new media (e.g., Internet) following the March 2011 Japan Earthquake. However, First et al. (2020) found that social media use was associated with higher levels of stress and depression than traditional media use following the COVID-19 pandemic. Therefore, the associations between media exposure forms and acute stress responses deserve further exploration.

### Media exposure content and acute stress responses

Disaster media coverage often includes various content, which may have distinct relations with acute stress responses. Previous studies have demonstrated the complex relationships between different media exposure content and psychological outcomes (Ahern et al., 2002; Holman et al., 2019). A study about Typhoon Hato found that viewing drowning victims and viewing residents’ emotional reactions were positively associated with PTSD, whereas viewing more information related to the storm itself and viewing images of people being heroic were protective factors for PTSD (Hall et al., 2019). Chao et al. (2020) also revealed that viewing stressful content was associated with more negative affect and depressive symptoms, whereas viewing people being heroic, speeches from experts and knowledge of the disease and prevention were related to more positive affect. However, another study found that viewing heroic images had no effects on mental health after the 9/11 terrorist attacks (Saylor et al., 2003). Additionally, some researchers found that the characteristics of media exposure content are related to psychological distress during the disaster-related events (Houston, Spialek & First, 2018; Ranjit et al., 2020). Media exposure to potentially distressing or frightening content may be associated with negative mental and physical responses. For example, individuals who reported more frequent exposure to distressing graphic content on television (e.g., people falling from the buildings) showed a higher risk of PTSD following the 9/11 terrorist attacks (Ahern et al., 2002). Such findings highlighted the important role of media exposure content after traumatic events. Currently, the associations between media exposure content and acute stress responses following the pandemic events should be further explored based on Chinese social context.

### Individual characteristics and acute stress responses

Individual characteristics may be associated with stress-related responses after exposure to traumatic events, even in an indirect way (Yeung et al., 2016). Previous research has revealed that sex (Baschnagel et al., 2009), age (Silver et al., 2013), and marital status (Ford, Adams & Dailey, 2007) can predict psychological health following traumatic events. Meanwhile, direct trauma exposure, history of mental health, recent adverse life events and collective trauma experiences may be associated with higher risks of psychological problems after disaster events (Garfin, Holman & Silver, 2019; Holman, Garfin & Silver, 2014; Yeung et al., 2016). However, it is unclear to what extent these individual characteristics relate to acute stress responses during the early COVID-19 outbreak in China.

### The present study

Following the above, the present study aimed to examine the relationships between the media exposure to COVID-19 (i.e., media exposure time, media exposure forms and media exposure content) and acute stress responses (i.e., continuous acute stress symptom scores and probable ASD) during the early COVID-19 outbreak in China. The associations between individual characteristics and acute stress responses were also investigated. We hypothesized that (1) longer media exposure time was associated with higher acute stress and a greater risk of probable ASD, (2) different media exposure forms had distinct relations to acute stress and probable ASD, (3) different media exposure content had distinct relations to acute stress and probable ASD, and (4) individual characteristics, including sex, age, marital status, direct exposure extent to COVID-19, history of mental health, recent adverse life events and collective trauma experiences were significantly related to acute stress responses.

## Materials and Methods

### Participants and procedure

This web-based cross-sectional survey was conducted from February 3 to February 10, 2020, during the early COVID-19 outbreak in China. A total of 1,587 participants from 32 provincial administrative divisions in mainland China were recruited through social media (e.g., WeChat, Tencent QQ) by sending electronic questionnaires. After removing participants who gave uniform answers to all items in the questionnaire (*n* = 121, 7.62%) and those who were infected with COVID-19 (*n* = 16, 1.01%), the final sample included 1,450 participants (91.37%). Participants were required to provide electronic informed consent prior to their participation. The participation was voluntary and survey responses were anonymous. The School of Psychology, Guizhou Normal University granted Ethical approval to carry out the study within its facilities (Ethical Application Ref: GNU-XLXY-2020024). All procedures performed in studies involving human participants were in accordance with the 1964 Helsinki declaration and its later amendments or comparable ethical standards.

### Measures

### Direct exposure extent to COVID-19

Direct exposure extent to COVID-19 was measured by three dichotomous items separately. Respondents were asked whether they lived in Hubei Province, and whether someone close to them lived in Hubei Province in the latest week, in that Hubei Province was the epicenter of the COVID-19 pandemic in China during that time. Another dichotomous question asked respondents whether someone close to them was diagnosed with COVID-19.

### History of mental illness

History of mental illness was assessed by asking participants whether they had ever been diagnosed with mental illness (e.g., anxiety disorder, depression). According to the reports of subjects, history of mental illness was coded 0 (none) or 1 (one or more mental illnesses) for final analyses. Similar categorizations have been used in previous studies (e.g.,  Holman, Garfin & Silver, 2014; Garfin et al., 2014).

### Recent adverse life events

Resent adverse life events were evaluated by using an 8-item checklist (e.g., death of relatives, divorce of oneself or parents, serious accidents). Participants reported whether they had experienced any of 8 common adverse life events in the last three years. The adverse life events were coded 0 (none) or 1 (one or more adverse life events) based on the accumulated number of events.

### Prior collective trauma exposure

Participants were asked about prior exposure to the 2008 Wenchuan Earthquake and SARS in 2003 by 2 items separately. First, participants reported their experiences about the 2008 Wenchuan Earthquake on a 5-point scale (1 = never heard of it, 2 = knew about it only on television, 3 = slight tremor where I lived, 4 = moderate tremor where I lived, 5 = strong tremor where I lived). The degree of trauma caused by the 2008 Wenchuan Earthquake was then assessed by a 5-point scale ranging from 1 (nil) to 5 (extremely severe). The total scores of 2 items were used to reflect the prior exposure to the 2008 Wenchuan Earthquake. Similarly, the exposure extent to SARS was measured by asking subjects to choose one from the five answers (1 = never heard of it, 2 = knew about it only on television, 3 = far from the pandemic center, 4 = close to the pandemic center, 5 = being in the pandemic center). The degree of trauma caused by SARS was evaluated by using the same 5-point scale as the 2008 Wenchuan Earthquake.

### Media exposure to COVID-19

Media exposure to COVID-19 was consisted of three sections, including media exposure time, media exposure forms and media exposure content. Media exposure time was assessed by asking participants how many hours in total per day (0–24 h) they spent engaging with information about COVID-19 from all media sources. Four questions were used to examine the amount of time spent on different forms of media exposure, including traditional media (e.g., newspaper, radio, television), social media (e.g., WeChat, Tencent QQ, Sina Weibo), online news (e.g., Toutiao, Xinhuanet, Sohu), short video application (e.g., Tik Tok, Kuaishou). For example, how many hours per day did you spend on social media (e.g., WeChat, Tencent QQ, Sina Weibo) to know about COVID-19 in the latest week. Six items were used to assess the media exposure content that respondents viewed by various media. For example, how often did you view the content about heroic deeds in fighting against COVID-19 (range from 0 = never to 4 = always). Similar questions have been previously used successfully to assess the relationships between media exposure to traumatic events and mental health outcomes (Rodgers et al., 2012; Holman, Garfin & Silver, 2014; Hall et al., 2019).

### Acute stress

Stanford Acute Stress Reaction Questionnaire (SASRQ) is usually used to measure acute stress and acute stress disorders (ASD) (Cardeña et al., 2000). The Chinese version of SASRQ was revised by Jia & Hou (2009) through standard translation and back-translation procedure. Many empirical results have showed that the Chinese version of SASRQ has a good reliability and validity (Hu et al., 2011; Li & Chen, 2015; Liu, Zhou & Li, 2019). In present study, some items were modified to ensure that the scale could be suitable to assess COVID-19-related acute stress responses by reference to previous research (Holman, Garfin & Silver, 2014; Holman et al., 2019; Thompson et al., 2019). An example item is “The COVID-19 pandemic made it difficult for me to perform work or other things I needed to do”. The SASRQ is a self-report measure with 30 items including dissociation (10 items), reexperiencing of trauma (6 items), avoidance (6 items), anxiety and hyperarousal (6 items), and impairment in functioning (2 items). The measure uses a 6-point Likert-type scale from 0 (not experienced) to 5 (very often experienced), with the total scores ranging from 0 to 150. The SASRQ is mainly used to assess the severity of acute stress, with higher scores indicating greater symptomatology. Besides, the SASRQ is a reliable aided diagnostic tool for ASD symptoms. Based on the diagnostic criteria B, C, D, and E for ASD in DSM-5 (American Psychiatric Association, 2013), respondents who met all four criteria were classified as probable ASD. Specifically, one respondent with at least 3 dissociation, 1 reexperiencing of trauma, 1 avoidance, and 1 anxiety and hyperarousal was assumed to meet criteria B. A single item with scores ≥ 3 was considered as the presence of a clinical symptom. Notably, participants were not classified as identified ASD in that most of them did not meet criteria A (direct exposure to traumatic events) for ASD in DSM-5 (American Psychiatric Association, 2013). The Cronbach’s *α* in the current study was 0.96.

### Statistical analysis

Statistical analyses were performed on SPSS Version 25.0. Data was described using mean, standard deviation, frequency and percentage. Independent *t*-test and one-way ANOVA were used to compare the mean scores of continuous acute stress symptom among different groups. Pearson’s chi-square test and Fisher’s exact test were used to analyze the differences in categorical variables about the dichotomous indices of probable ASD.

Multiple linear regression analyses and multiple logistic regression analyses were then conducted to assess the significant predictors of continuous acute stress symptom scores and dichotomous indices of probable ASD, respectively. The two kinds of regression models kept the same stepwise sequences of variables. The variables entered into the two models in the same sequence. Basic demographic variables (age, gender, marital status and place of residence) were entered in Step 1, followed by the direct exposure extent to COVID-19 in Step 2. Prior collective trauma exposure, history of mental illness and recent adverse life events were entered in Step 3. Finally, we added media exposure in Step 4. Media exposure time, media exposure forms and media exposure content were analyzed separately (Model 4a, 4b and 4c). All statistical tests were two-sided, with a significance level of *p* < 0.05.

## Results

### Participant characteristics

A total of 1,450 participants were included in current study. Among the participants, 464 (32%) were men and 986 (68%) were women, with a mean age of 28.15 years (*SD* = 8.52; range: 11–87 years), and 894 (61.66%) were single. Nearly half of the respondents lived in city (*n* = 693, 47.79%), 269 (18.55%) lived in town, and 488 (33.66%) lived in village. For living region, 131 (9.03%) lived in Hubei Province, and 1,319 (90.97%) lived in other provinces. More descriptive statistics are summarized in Table 1.

**Table 1:** Descriptive statistics and comparison of continuous acute stress symptom scores and probable ASD on demographic variables.

Variables	N	Continuous acute stress symptom scores	P	NO probable ASD (*N* = 1,358)	Probable ASD (*N* = 92)	X^2^	*P*
		M ± SD		*N* (%)	*N* (%)		
Gender							
Male	464	21.00 ± 23.60	0.036	436(94.0%)	28(6.0%)	0.11	0.739
Female	986	23.71 ± 22.59		922(93.5%)	64(6.5%)		
Marital status							
Single	894	23.76 ± 22.84	0.026	835(93.4%)	59(6.6%)	Fisher’s exact	0.167
Married	526	20.94 ± 22.61		497(94.5%)	29(5.5%)		
Divorced or widowed	30	29.13 ± 29.21		26(86.7%)	4(13.3%)		
Place of residence							
City	693	23.15 ± 23.39	0.876	650(93.8%)	43(6.2%)	0.06	0.970
Town	269	22.75 ± 22.51		252(93.7%)	17(6.3%)		
Village	488	22.46 ± 22.57		456(93.4%)	32(6.6%)		
Living region							
Hubei Province	131	29.20 ± 28.79	0.008	118(90.1%)	13(9.9%)	3.10	0.078
Non-Hubei Province	1,319	22.21 ± 22.19		1240(94.0%)	79(6.0%)		
Someone close to them lived in Hubei Province							
No	1,075	21.51 ± 22.49	<0.001	1008(93.8%)	67(6.2%)	0.09	0.767
Yes	375	26.66 ± 23.81		350(93.3%)	25(6.7%)		
Someone close to them was diagnosed with COVID-19							
No	1,360	22.21 ± 22.05	0.004	1281(94.2%)	79(5.8%)	10.59	0.001
Yes	90	32.52 ± 32.31		77(85.6%)	13(14.4%)		
History of mental illness							
No	1,371	21.56 ± 21.77	<0.001	1301(94.9%)	70(5.1%)	65.02	<0.001
Yes	79	45.22 ± 30.32		57(72.2%)	22(27.8%)		
Adverse life events							
No	927	20.34 ± 21.99	<0.001	886(95.6%)	41(4.4%)	15.98	<0.001
Yes	523	27.29 ± 23.92		472(90.2%)	51(9.8%)		
Media exposure time (hours per day)							
<3	322	16.21 ± 18.30	<0.001	313(97.2%)	9(2.8%)	17.09	<0.001
3-8	712	22.46 ± 22.30		671(94.2%)	41(5.8%)		
>8	416	28.64 ± 25.66		374(89.9%)	42(10.1%)		

### Comparison of continuous acute stress symptom scores on demographic variables

The mean total continuous acute stress symptom score was 22.85 (*SD* = 22.94). As shown in Table 1, *t*-tests showed that there were significant differences in acute stress scores between different genders (*p* < 0.05) and between different living regions (*p* < 0.01). Participants with someone close to them living in Hubei or diagnosed with COVID-19 reported significantly higher acute stress scores (both *ps* < 0.01). Similarly, those who had histories of mental illness, or experienced more adverse life events showed significantly higher acute stress scores (both *ps* < 0.001). One-way ANOVAs indicated that there were significant differences in acute stress scores among different marital status (*p* < 0.05), whereas no significant differences were found in acute stress scores among different places of residence (*p* > 0.05). In terms of media exposure time, participants who spent more time on viewing information about COVID-19 tended to report higher acute stress scores (*p* < 0.001).

### Comparison of probable ASD on demographic variables

According to the criteria B, C, D, and E for ASD in DSM-5, the overall prevalence of probable ASD was 6.3% (*n* = 92). Table 1 displays the comparison of probable ASD on demographic variables. Chi-square tests and Fisher’s exact tests revealed that there were no significant differences in gender, marital status, place of residence, living region and whether someone close to them lived in Hubei Province (all *ps* > 0.05). Participants with someone close to them diagnosed with COVID-19, having histories of mental illness or more adverse life events were more likely to experience probable ASD (all *ps* < 0.01). Furthermore, those with longer media exposure time were more likely to report probable ASD (*p* < 0.001).

### Factors predicting continuous acute stress symptom scores

The multiple linear regression analyses are presented in Tables 2 and 3. Model 1 indicated that there were no associations between age, gender, marital status, place of residence and acute stress (all *ps* > 0.05). In Model 2, living in Hubei Province, someone close to them living in Hubei Province and someone close to them diagnosed with COVID-19 were predictors of high acute stress (all *ps* < 0.05). In Model 3, having histories of mental illness, experiencing more adverse life events, prior exposure to the 2008 Wenchuan Earthquake, and prior exposure to SARS were all significantly associated with higher acute stress (all *ps* < 0.01). For the media exposure time, longer time was associated with higher levels of acute stress (both *ps* < 0.01) (Model 4a). For the media exposure forms, social media was associated with higher acute stress (*p* < 0.001) (Model 4b). For the media exposure content, viewing the situation of infected patients was associated with higher acute stress (*p* < 0.01) (Model 4c). Moreover, being female was significantly associated with higher acute stress in Model 2, Model 3, Model 4a, Model 4b and Model 4c (all *ps* < 0.05).

### Factors predicting probable ASD

The multiple logistic regression models are shown in Tables 4 and 5. Model 1 and Model 2 were not significant. However, participants with someone close to them diagnosed with COVID-19 had higher odds of probable ASD in Model 2 (*p* < 0.01). In Model 3, having histories of mental illness, experiencing more adverse life events, prior exposure to the 2008 Wenchuan Earthquake were all significantly associated with increased odds of probable ASD (all *ps* < 0.05). Table 5 mainly presents the associations between media exposure time, media exposure forms, media exposure content and probable ASD, separately. In Model 4a, more than 8 h of media exposure were significantly associated with higher odds of probable ASD (*p* < 0.01). In Model 4b, social media was significantly associated with higher odds of probable ASD (*p* < 0.05). In Model 4c, viewing latest news about pandemic data was significantly associated with lower odds of probable ASD (*p* < 0.05). Furthermore, living in Hubei Province and someone close to them diagnosed with COVID-19 were both significantly associated with higher odds of probable ASD in Model 3, Model 4a, Model 4b and Model 4c (all *ps* < 0.05).

**Table 2:** Multiple linear regression Model 1, 2 and 3 predicting continuous acute stress symptom scores.

**Predictor variable**	**Model 1**			**Model 2**			**Model 3**		
	*β*	***95%CI***	***p***	*β*	***95%CI***	***p***	*β*	***95%CI***	***p***
Age	−0.04	−0.32-0.10	0.295	−0.04	−0.32-0.10	0.314	−0.06	−0.36-0.05	0.134
Gender									
Female (ref. male)	0.05	-0.11-4.99	0.060	0.06	0.25-5.31	0.031	0.07	0.95-5.77	0.006
Marital status									
Single (reference group)									
Married	−0.04	−5.66-1.67	0.285	−0.06	−6.32-0.96	0.149	−0.06	−6.16-0.76	0.126
Divorced or widowed	0.04	−3.04-15.04	0.193	0.04	−2.89-15.03	0.184	0.02	−4.96-12.03	0.415
Place of residence									
City (reference group)									
Town	−0.02	−4.75-1.89	0.398	−0.01	−3.61-3.02	0.861	0.00	−3.10-3.20	0.975
Village	−0.04	−4.94-0.84	0.165	−0.02	−3.93-1.84	0.477	−0.01	−3.29-2.20	0.698
Living region									
Hubei Province (ref. Non-Hubei Province)				0.06	0.34-9.30	0.035	0.08	1.89-10.39	0.005
Someone close to them lived in Hubei Province									
Yes (ref. no)				0.06	0.34-6.19	0.029	0.04	−0.85-4.73	0.173
Someone close to them was diagnosed with COVID-19									
Yes (ref. no)				0.09	3.15-13.27	0.001	0.07	1.77-11.38	0.007
History of mental illness									
Yes (ref. no)							0.21	15.90-25.79	<0.001
Adverse life events									
Yes (ref. no)							0.09	1.73- 6.43	0.001
Prior exposure to the 2008 Wenchuan Earthquake							0.16	2.40- 4.64	<0.001
Prior exposure to SARS							0.09	0.89-3.63	0.001

**Notes.**

*N* = 1,450. All models were significantModel 1: *F*(6,1,443) = 2.33, *p* < 0.05; Model 2: *F*(9,1,440) = 5.16, *p* < 0.001; Model 3: *F*(13,1,436) = 16.91, *p* < 0.001

CI confidence interval

**Table 3:** Multiple linear regression Model 4a, 4b and 4c predicting continuous acute stress symptom scores.

**Predictor variable**	**Model 4a**			**Model 4b**			**Model 4c**		
	*β*	***95%CI***	***p***	*β*	***95%CI***	***p***	*β*	***95%CI***	***p***
Age	−0.06	−0.35-0.05	0.136	−0.05	−0.33-0.07	0.193	−0.06	−0.36-0.05	0.137
Gender									
Female (ref. male)	0.06	0.62-5.39	0.013	0.06	0.37-5.16	0.024	0.06	0.55-5.42	0.016
Marital status									
Single (reference group)									
Married	−0.05	−5.93-0.92	0.152	−0.05	−5.81-1.04	0.171	−0.06	−6.21-0.70	0.117
Divorced or widowed	0.02	−4.83-11.98	0.404	0.02	−4.68-12.11	0.386	0.02	−5.17-11.80	0.444
Place of residence									
City (reference group)									
Town	0.01	−2.80-3.43	0.843	0.01	−2.29-3.95	0.599	0.00	−3.14-3.15	0.999
Village	−0.01	−3.16-2.28	0.752	0.00	−2.76-2.73	0.993	−0.01	−3.34-2.17	0.677
Living region									
Hubei Province (ref. Non-Hubei Province)	0.07	1.34-9.75	0.010	0.08	2.04-10.45	0.004	0.07	1.45-9.95	0.009
Someone close to them lived in Hubei Province									
Yes (ref. no)	0.04	−0.78-4.74	0.160	0.03	−1.07-4.45	0.229	0.04	−0.78-4.80	0.157
Someone close to them was diagnosed with COVID-19									
Yes (ref. no)	0.06	1.43-10.94	0.011	0.06	1.18-10.66	0.014	0.07	1.50-11.08	0.010
History of mental illness									
Yes (ref. no)	0.20	15.76-25.54	<0.001	0.20	15.07-24.86	<0.001	0.20	15.54-25.50	<0.001
Adverse life events									
Yes (ref. no)	0.08	1.39-6.04	0.002	0.08	1.67-6.30	0.001	0.09	1.86-6.56	<0.001
Prior exposure to the 2008 Wenchuan Earthquake	0.14	1.89-4.13	<0.001	0.14	1.92-4.15	<0.001	0.15	2.26-4.50	<0.001
Prior exposure to SARS	0.09	0.84-3.54	0.002	0.08	0.66-3.36	0.004	0.08	0.74-3.48	0.003
Media exposure time (hours per day)									
<3 (reference group)									
3-8	0.10	1.60-7.26	0.002						
>8	0.18	5.92-12.23	<0.001						
Media exposure forms									
Traditional media				−0.04	−1.27-0.22	0.167			
Social media				0.15	0.87-1.93	<0.001			
Online news				0.06	−0.02-1.51	0.055			
Short video application				0.03	−0.44-1.52	0.280			
Media exposure contents									
Latest news about pandemic data							−0.02	−7.90-3.97	0.517
Progress on vaccine development							0.04	−0.58-5.25	0.117
Influence on society							−0.04	−4.57-0.98	0.205
Influence on daily life							0.03	−1.30-4.36	0.290
The situation of infected patients							0.09	1.15-6.65	0.006
Heroic deeds							−0.02	−3.77-1.64	0.440

**Notes.**

*N* = 1,450. All models were significant. Model 4a: *F*(15,1,434)=17.11, *p* < 0.001; Model 4b: *F*(17,1,432)=16.24, *p* < 0.001; Model 4c: *F*(19,1,430)=12.44, *p* < 0.001.

CI confidence interval

**Table 4:** Multiple logistic regression Model 1, 2 and 3 predicting probable ASD.

**Predictor variable**	**Model 1**			**Model 2**			**Model 3**		
	*OR*	***95%CI***	***p***	*OR*	***95%CI***	***p***	*OR*	***95%CI***	***p***
Age	0.99	0.95-1.03	0.220	0.99	0.95-1.03	0.611	0.99	0.95-1.04	0.645
Gender									
Female (ref. male)	1.05	0.66-1.67	0.838	1.09	0.68-1.74	0.715	1.22	0.74-1.99	0.441
Marital status									
Single (reference group)									
Married	0.95	0.48-1.87	0.885	0.90	0.45-1.79	0.758	0.87	0.43-1.77	0.699
Divorced or widowed	2.59	0.73-9.21	0.140	2.69	0.74-9.78	0.133	1.94	0.49-7.67	0.347
Place of residence									
City (reference group)									
Town	0.99	0.55-1.81	0.979	1.14	0.62-2.10	0.683	1.29	0.67-2.45	0.446
Village	1.02	0.61-1.70	0.954	1.11	0.65-1.88	0.706	1.32	0.76-2.30	0.321
Living region									
Hubei Province (ref. Non-Hubei Province)				1.70	0.83-3.49	0.148	2.32	1.10-4.86	0.026
Someone close to them lived in Hubei Province									
Yes (ref. no)				1.26	0.73-2.20	0.409	1.50	0.84-2.67	0.170
Someone close to them was diagnosed with COVID-19									
Yes (ref. no)				2.85	1.43-5.69	0.003	2.40	1.15-5.03	0.020
History of mental illness									
Yes (ref. no)							6.10	3.37- 11.05	<0.001
Adverse life events									
Yes (ref. no)							1.74	1.10- 2.75	0.017
Prior exposure to the 2008 Wenchuan Earthquake							1.51	1.25-1.82	<0.001
Prior exposure to SARS							1.23	0.97-1.56	0.095

**Notes.**

*N* = 1,450. Model 3 was significant: Wald *χ*^2^(6, *N* = 1,450)=85.76, *p* < 0.001

CI confidence interval OR odds ratio

**Table 5:** Multiple logistic regression Model 4a, 4b and 4c predicting probable ASD.

**Predictor variable**	**Model 4a**			**Model 4b**			**Model 4c**		
	*OR*	***95%CI***	***p***	*OR*	***95%CI***	***p***	*OR*	***95%CI***	***p***
Age	0.99	0.95-1.04	0.704	1.00	0.95-1.04	0.832	0.99	0.95-1.04	0.705
Gender									
Female (ref. male)	1.18	0.72-1.95	0.506	1.18	0.71-1.96	0.523	1.27	0.77-2.11	0.352
Marital status									
Single (reference group)									
Married	0.89	0.43-1.82	0.743	0.89	0.43-1.84	0.744	0.85	0.42-1.73	0.654
Divorced or widowed	2.14	0.55-8.30	0.273	2.11	0.54-8.22	0.284	1.93	0.49-7.66	0.352
Place of residence									
City (reference group)									
Town	1.34	0.70-2.56	0.380	1.43	0.74-2.76	0.282	1.28	0.67-2.46	0.456
Village	1.38	0.79-2.42	0.257	1.35	0.77-2.39	0.298	1.39	0.80-2.44	0.246
Living region									
Hubei Province (ref. Non-Hubei Province)	2.23	1.06-4.67	0.034	2.33	1.10-4.92	0.028	2.14	1.02-4.52	0.045
Someone close to them lived in Hubei Province									
Yes (ref. no)	1.53	0.85-2.75	0.152	1.53	0.85-2.75	0.160	1.43	0.80-2.55	0.229
Someone close to them was diagnosed with COVID-19									
Yes (ref. no)	2.48	1.18-5.20	0.016	2.41	1.14-5.11	0.021	2.35	1.12-4.95	0.024
History of mental illness									
Yes (ref. no)	6.11	3.35-11.14	<0.001	6.03	3.28-11.11	<0.001	5.77	3.15-10.57	<0.001
Adverse life events									
Yes (ref. no)	1.69	1.07-2.68	0.025	1.73	1.09- 2.74	0.020	1.84	1.16-2.91	0.010
Prior exposure to the 2008 Wenchuan Earthquake	1.45	1.20-1.76	<0.001	1.44	1.18-1.75	<0.001	1.53	1.26-1.84	<0.001
Prior exposure to SARS	1.23	0.97-1.56	0.095	1.19	0.94-1.52	0.157	1.23	0.97-1.56	0.090
Media exposure time (hours per day)									
<3 (reference group)									
3-8	1.67	0.78-3.60	0.189						
>8	2.86	1.32- 6.16	0.008						
Media exposure forms									
Traditional media				1.01	0.89-1.15	0.829			
Social media				1.10	1.00-1.21	0.041			
Online news				1.13	1.00-1.27	0.055			
Short video application				1.08	0.91-1.28	0.372			
Media exposure contents									
Latest news about pandemic data							0.36	0.14-0.90	0.029
Progress on vaccine development							0.95	0.52-1.73	0.854
Influence on society							1.04	0.58-1.87	0.887
Influence on daily life							1.13	0.62-2.06	0.698
The situation of infected patients							1.43	0.81-2.53	0.213
Heroic deeds							0.84	0.48-1.46	0.531

**Notes.**

*N* = 1,450. All models were significant. Model 4a: Wald *χ*^2^(15,*N* = 1,450) = 95.30, *p* < 0.001; Model 4b: Wald *X*^2^(17, *N* = 1,450)=103.73, *p* < 0.001; Model 4c: Wald *χ*^2^(19, *N* = 1,450)=92.12, *p* < 0.001.

CI confidence interval OR odds ratio

## Discussion

To our knowledge, this is one of the first studies to examine the relations between media exposure to COVID-19 and acute stress responses during the early COVID-19 outbreak in China. Our results showed that 6.3% of participants were identified as probable ASD, and the widespread media coverage related to the COVID-19 pandemic was associated with acute stress responses. Supporting our hypotheses, media exposure time, media exposure forms and media exposure content were all associated with acute stress responses after controlling for basic demographics, direct exposure extent to COVID-19, history of mental illness, recent adverse life events and prior collective trauma exposure. These findings are congruent with previous studies demonstrating the important links between media exposure to traumatic events and mental health (Chao et al., 2020; Hall et al., 2019; Holman, Garfin & Silver, 2014).

### Media exposure time and acute stress responses

Participants who spent more time on viewing information about COVID-19 were significantly associated with higher acute stress levels and odds of probable ASD. The results are in accordance with prior studies showing that longer trauma-related media exposure could predict higher acute stress symptomatology (Goodwin et al., 2015; Holman, Garfin & Silver, 2014; Thompson et al., 2019; Silver et al., 2013). During the early COVID-19 outbreak, the continuous spread of the pandemic caused social isolation of an entire nation. Under this situation, people also had a great need for information to make sense of the pandemic and reduce potential risks and uncertainty. Generally, media was the main source of pandemic-related information for the majority of people during the COVID-19 outbreak in China. However, long term media exposure is likely to reinforce rumination and intrusive thoughts, activate fear circuitry (Bourne, Mackay & Holmes, 2013; Holman, Garfin & Silver, 2014; Hong et al., 2020), amplify the perception of risk (Chao et al., 2020; Garfin, Silver & Holman, 2020), even enhance autonomic activation and affect physiologic systems (Watkins, 2008; Brosschot, 2010; Gerin et al., 2012), all of which may lead to the increase of acute stress and the development of probable ASD. This is also consistent with several findings that trauma-related media exposure could predict negative psychological symptoms, such as PTSD (Palgi, Shrira & Hoffman, 2017), insomnia (Goodwin, Lemola & Ben-Ezra, 2018), anxiety, depression, and stress (Chao et al., 2020; Gao et al., 2020). To the extent to which mass media coverage of disasters may in and of itself represent an “exposure” (Bernstein et al., 2007), our results may help explain that mass media can be recognized as a particular risk factor following trauma (Goodwin et al., 2013; Thompson et al., 2019). In addition, the associations between media exposure and mental health have been reported in other community crises, such as the 9/11 terrorist attacks (Silver et al., 2013), the 2008 Wenchuan Earthquake (Yeung et al., 2016) and Typhoon Hato (Hall et al., 2019), which may reveal a “cross-disaster” phenomenon that media exposure can predict negative psychological outcomes in different traumatic events.

### Media exposure forms and acute stress responses

Among the various media exposure forms, only social media was significantly associated with higher acute stress and the odds of probable ASD. The result is consistent with the previous findings that social media users had more psychological problems compared with traditional media users during the COVID-19 pandemic (Chao et al., 2020; First et al., 2020). Social media was usually regarded as the most extensively used medium for acquiring information about COVID-19 without time and site limits. However, repeated social media exposure may increase community stress and particularly induce negative affect due to the nature of uncontrolled information quality (Resnyansky, 2014; Van der Meer & Verhoeven, 2013). This finding is also in line with the emotional contagion hypothesis (Kramer, Guillory & Hancock, 2014). Within the dynamic disaster context, information is easier to be shared and viewed by social media (Chao et al., 2020). However, these information potentially contains a variety of negative emotions, which may result in increased worry or anxiety by interpersonal communication. Likewise, individuals with more time spending on the social media are inclined to take dysfunctional emotion regulation strategies (Hatfreld, Cacioppo & Rapson, 1993; Monfort & Afzali, 2017). As a result, they are more vulnerable to experience psychological distress. Our results suggest that it is important to note the appropriate use of social media following collective trauma. Governments and other relevant agencies should implement mental health interventions to reduce the negative psychological effects.

### Media exposure content and acute stress responses

Multiple linear regression analyses indicated that viewing the infected patients’ situation was associated with higher acute stress. According to the emotional contagion hypothesis, emotional contagion is an interactive process among individuals (Du, Fan & Feng, 2011), which means that negative emotions could be contagious to each other. Similarly, individuals frequently viewing the infected patients’ situation might be influenced by the negative emotions of infected patients. The result supports a previous study following the Typhoon Hato in Macao indicating that viewing residents’ emotional reaction was associated with higher risks of PTSD (Hall et al., 2019). In addition, this finding is in line with research demonstrating the links between distressing media exposure content and worse psychological health (Houston, Spialek & First, 2018; Ranjit et al., 2020). However, multiple logistic regression analyses suggested viewing latest news about pandemic data was significantly associated with lower odds of probable ASD. It is plausible given that previous studies have revealed a positive effect of media exposure following disasters (Tandoc & Takahashi, 2017; Chao et al., 2020). In fact, the latest news about pandemic data mainly came from official governments during the COVID-19 outbreak in China, which was accurate and credible for most people. Uncertainty reduction theory suggests that people tend to seek information about the potential threat to reduce anxiety (Boyle et al., 2004). Hence, reliable and timely information about COVID-19 may facilitate the reduction of uncertainty. Similarly, such information-seeking behaviors may reduce psychological distress and increase the sense of security by facilitating deliberate rumination (Yoshida et al., 2016). Thus, official governments and competent departments should timely release accurate coronavirus-related information to relieve the stress-related symptoms of the public. Interestingly, no associations were found between viewing heroic deeds and acute stress responses in current study. This finding is inconsistent with one previous study that has shown the positive relation between viewing people being heroic and positive affect during the COVID-19 outbreak (Chao et al., 2020). Thus, much more research is needed to clarify the complex relationships between viewing heroic deeds and psychological distress.

### Individual characteristics and acute stress responses

Being females was related to higher acute stress, which supports most previous studies demonstrating that females generally have more serious psychological symptoms than males following the disaster-related events (Baschnagel et al., 2009; Xiao et al., 2020). However, regression analysis results documented that there were no associations between age and acute stress or probable ASD. Actually, age differences in the psychological impacts of COVID-19 pandemic are controversial in previous studies (Tian et al., 2020; Chao et al., 2020). Therefore, further research is warranted to explore the causes of age differences. Contrary to the previous studies (Ford, Adams & Dailey, 2007; Tian et al., 2020), there were no significant differences in marital status. This indicated that individuals with different marital status may experience similar acute stress and negative emotions during the pandemic.

With regard to direct exposure extent to COVID-19, regression models suggested that individuals living in Hubei Province presented higher acute stress and probable ASD than other regions, which supports one previous empirical study (Xiao et al., 2020). Considering that Hubei Province was at the center of the COVID-19 pandemic, it is not surprising that individuals living in Hubei Province had more acute stress responses. Meanwhile, we found that someone close to them diagnosed with COVID-19 was associated with higher acute stress and probable ASD. It is possible that the COVID-19 infection of a close relative or close friend may induce fear and uncertainty for the virus, thus leading to the increase of acute stress responses. Moreover, consistent with criteria A for ASD in DSM-5 (American Psychiatric Association, 2013), these findings further indicated that direct exposure extent could be an important risk factor to predict acute stress responses during the COVID-19 outbreak.

The current study also found that history of mental illness and recent adverse life events were positively associated with acute stress and probable ASD. These results are congruent with previous research suggesting that prior negative life experiences were significantly related to current mental health outcomes (Garfin, Holman & Silver, 2019; Mullett-Hume et al., 2008; Sweeting et al., 2020). It is interesting that previous exposure to the 2008 Wenchuan Earthquake was associated with high acute stress and probable ASD. This result supports previous research highlighting that exposure to similar events may render some individuals more vulnerable to the negative effects of subsequent traumatic events (McLaughlin et al., 2010; Holman, Garfin & Silver, 2014). Thus, media exposure may trigger suppressed traumatic memories from prior traumatic experiences, which in turn elicit stress-related responses. However, prior exposure to SARS was only associated with high acute stress but not probable ASD. This may be due to that SARS broke out in 2003, so long ago that the memories of it were no longer clear, and the psychological impacts have also diminished over time.

### Limitations

Several limitations should be mentioned. First, we collected data in a shorter timeframe, and no assessments occurred during the follow-up, which may prevent us from detecting the changes of acute stress responses over time. Second, responses were self-reported, which were potentially subject to recall bias. Acute stress responses were only assessed by online questionnaires instead of clinical interviews. However, there is controversy in the field as to what characterizes the diagnostic criteria for ASD. Thus, future studies should further explore the effects of media exposure on the prevalence of ASD by using other evaluation criteria of ASD following pandemic events. Third, the current study was cross-sectional, which limited causal inference. Therefore, future longitudinal studies in this field are needed. Fourth, although large sample, the survey was online based. Thus, the selection bias may have affected the results, which can limit the representativeness of our sample. Moreover, the sample in our study only included Chinese citizens. It is uncertain to what extent the results generalize to samples from other countries and regions. Fifth, the current study only assessed acute stress responses without studying the effect of media exposure to COVID-19 on PTSD. However, some people may suffer from PTSD over time after the outbreak of COVID-19. Thus, future studies are encouraged to further explore the associations between pandemic-related media exposure and PTSD.

## Conclusions

The current study found that media exposure to COVID-19 was significantly related to acute stress and probable ASD during the early COVID-19 outbreak in China. Specifically, longer media exposure time and the use of social media were associated with higher acute stress and probable ASD. Viewing the situation of infected patients was positively related to acute stress, whereas viewing the latest news about pandemic data was negatively correlated with probable ASD. In addition, being females, living in Hubei Province, someone close to them diagnosed with COVID-19, history of mental illness, recent adverse life events and previous collective trauma exposure were all positively associated with acute stress responses. The current results may contribute to the overall literature by providing evidence on the relationships between indirect media exposure and psychological health after disasters. Future studies should explore the long-term psychological impacts of disaster-related media exposure and the mechanisms underlying the associations to reduce the corresponding psychological trauma.

##  Supplemental Information

10.7717/peerj.11407/supp-1 Supplemental Information 1 Questionnaires in Chinese and English

10.7717/peerj.11407/supp-1 Supplemental Information 2 Supplementary Files All the original data of the questionnaires.

## Additional Information and Declarations

### Data Availability

The following information was supplied regarding data availability: The raw measurements are available in the Supplemental File.
