# Supplementary material for: Media exposure predicts acute stress and probable acute stress disorder during the early COVID-19 outbreak in China
Source: PeerJ. 2021 May 10;9:e11407. doi: 10.7717/peerj.11407 (PMC8117928; doi:10.7717/peerj.11407)
Supplement: Supplemental Information 1 [file peerj-09-11407-s001.docx]

**Questionnaires**

**English version**

**Questionnaire 1:**

**Media exposure to COVID-19**

**Media exposure time**

1. How many hours in total per day (0-24 h) did you spend engaging with information about COVID-19 from all media sources in the latest week？

hour (s)

**Media exposure forms**

1. How many hours per day did you spend on traditional media (e.g., newspaper, radio, television) to know about COVID-19 in the latest week？ hour (s)
2. How many hours per day did you spend on social media (e.g., WeChat, Tencent QQ, Sina Weibo) to know about COVID-19 in the latest week？ hour (s)
3. How many hours per day did you spend on online news (e.g., Toutiao, Xinhuanet, Sohu) to know about COVID-19 in the latest week？ hour (s)
4. How many hours per day did you spend on short video application (e.g., Tik Tok, Kuaishou) to know about COVID-19 in the latest week？ hour (s)

**Media exposure content**

1. How often did you view the content on latest news about pandemic data？
2. Never B. Seldom C. Sometimes D. Often E. Always
3. How often did you view the content about the progress of COVID-19 on vaccine development？
4. Never B. Seldom C. Sometimes D. Often E. Always
5. How often did you view the content about the influence of COVID-19 on society?
6. Never B. Seldom C. Sometimes D. Often E. Always
7. How often did you view the content about the influence of COVID-19 on daily life?
8. Never B. Seldom C. Sometimes D. Often E. Always
9. How often did you view the content about the situation of infected patients?
10. Never B. Seldom C. Sometimes D. Often E. Always
11. How often did you view the content about heroic deeds in fighting against COVID-19?
12. Never B. Seldom C. Sometimes D. Often E. Always

**Questionnaire 2:**

**Stanford Acute Stress Reaction Questionnaire**

Below is a list of experiences people sometimes have during and after a stressful event. Please read each item carefully and decide how well it describes your experience during and immediately following the COVID-19 outbreak (usually in the latest week). Refer to this event in answering the items below. Use the 0-5 point scale shown below and circle the number that best describes your experience.

0------not experienced

1------very rarely experienced

2------rarely experienced

3------sometimes experienced

4------often experienced

5------very often experienced

| **Items** | **Levels** | | | | | |
| --- | --- | --- | --- | --- | --- | --- |
| 1. I had difficulty falling or staying asleep. | 0 | 1 | 2 | 3 | 4 | 5 |
| 2. I felt restless. | 0 | 1 | 2 | 3 | 4 | 5 |
| 3. I felt a sense of timelessness. | 0 | 1 | 2 | 3 | 4 | 5 |
| 4. I was slow to respond. | 0 | 1 | 2 | 3 | 4 | 5 |
| 5. I tried to avoid the feelings related to the COVID-19 pandemic. | 0 | 1 | 2 | 3 | 4 | 5 |
| 6. I had repeated distressing dreams of the COVID-19 pandemic. | 0 | 1 | 2 | 3 | 4 | 5 |
| 7. I would feel extremely upset if exposed to events that reminded me of an aspect of the COVID-19 pandemic. | 0 | 1 | 2 | 3 | 4 | 5 |
| 8. I would jump at the least thing. | 0 | 1 | 2 | 3 | 4 | 5 |
| 9. The COVID-19 pandemic made it difficult for me to perform work or other things I needed to do. | 0 | 1 | 2 | 3 | 4 | 5 |
| 10. I did not have the usual sense of who I am. | 0 | 1 | 2 | 3 | 4 | 5 |
| 11. I tried to avoid activities that reminded me of the COVID-19 pandemic. | 0 | 1 | 2 | 3 | 4 | 5 |
| 12. I felt hypervigilant or “on edge”. | 0 | 1 | 2 | 3 | 4 | 5 |
| 13. I experienced myself as though I were a stranger. | 0 | 1 | 2 | 3 | 4 | 5 |
| 14. I tried to avoid conversations about the COVID-19 pandemic. | 0 | 1 | 2 | 3 | 4 | 5 |
| 15. I had a bodily reaction when exposed to reminders of the COVID-19 pandemic. | 0 | 1 | 2 | 3 | 4 | 5 |
| 16. I had problems remembering important details about the COVID-19 pandemic. | 0 | 1 | 2 | 3 | 4 | 5 |
| 17. I tried to avoid thoughts about the COVID-19 pandemic. | 0 | 1 | 2 | 3 | 4 | 5 |
| 18. Things I saw looked different to me from how I know they really looked. | 0 | 1 | 2 | 3 | 4 | 5 |
| 19. I had repeated and unwanted memories related to the COVID-19 pandemic. | 0 | 1 | 2 | 3 | 4 | 5 |
| 20. I felt distant from my own emotions. | 0 | 1 | 2 | 3 | 4 | 5 |
| 21. I felt irritable or had outbursts of anger. | 0 | 1 | 2 | 3 | 4 | 5 |
| 22. I avoided contact with people who reminded me of the COVID-19 pandemic. | 0 | 1 | 2 | 3 | 4 | 5 |
| 23. I would suddenly act or feel as if the COVID-19 pandemic was breaking out again. | 0 | 1 | 2 | 3 | 4 | 5 |
| 24. My mind went blank. | 0 | 1 | 2 | 3 | 4 | 5 |
| 25. I had amnesia for large periods of the COVID-19 pandemic. | 0 | 1 | 2 | 3 | 4 | 5 |
| 26. The COVID-19 pandemic caused problems in my relationship with other people. | 0 | 1 | 2 | 3 | 4 | 5 |
| 27. I had difficulty concentrating. | 0 | 1 | 2 | 3 | 4 | 5 |
| 28. I felt estranged/detached from other people. | 0 | 1 | 2 | 3 | 4 | 5 |
| 29. I had a vivid sense that the COVID-19 pandemic was breaking out all over again. | 0 | 1 | 2 | 3 | 4 | 5 |
| 30. I tried to stay away from places that reminded me of the COVID-19 pandemic. | 0 | 1 | 2 | 3 | 4 | 5 |

**Questionnaire 3:**

**Individual characteristics**

1. Gender

A.Male B.Female

2. Age

years

3. Marital status

A.Single B.Married C.Divorced or widowed

4. Place of residence

A.City B.Town C.Village

**Direct exposure extent to COVID-19**

1. Did you live in Hubei Province in the latest week?

1. Yes B. No

2. Was there anyone close to you living in Hubei Province in the latest week?

1. Yes B. No

3. Was there anyone close to you diagnosed with COVID-19?

1. Yes B. No

**History of mental illness**

Have you ever been diagnosed with mental illness (e.g., anxiety disorder, depression)?

1. None B. One or more mental illnesses

**Recent adverse life events**

Please report whether you have experienced any of 8 common adverse life events in the last three years (other than COVID-19).

1. Death of relatives or friends

A. Yes B. No

2. Divorce of oneself or parents

A. Yes B. No

3. Serious diseases of oneself or family members

A. Yes B. No

4. Serious accidents of oneself or family members

A. Yes B. No

5. Serious natural disasters

A. Yes B. No

6. Serious family conflicts

A. Yes B. No

7. Criminal punishment

A. Yes B. No

8. Other serious traumatic events

A. Yes B. No

**Prior collective trauma exposure**

**Prior exposure to the 2008 Wenchuan Earthquake**

1. Please report your experiences about the 2008 Wenchuan Earthquake.

A. Never heard of it

B. Knew about it only on television

C. Slight tremor where I lived

D. Moderate tremor where I lived

E. Strong tremor where I lived

2. Please assess the degree of trauma caused by the 2008 Wenchuan Earthquake.

A. Nil B. Slight C. Moderate D. Severe E. Extremely severe

**Prior exposure to SARS**

1. Please report your experiences about SARS.

A. Never heard of it

B. Knew about it only on television

C. Far from the pandemic center

D. Close to the pandemic center

E. Being in the pandemic center

2. Please assess the degree of trauma caused by SARS.

A. Nil B. Slight C. Moderate D. Severe E. Extremely severe

**Chinese version**

**问卷1**

**媒体暴露问卷**

**媒体暴露时间**

最近一周，您平均每天通过媒体关注新冠肺炎疫情的时间有多长？（单位：小时，可具体到小数位，如：0.4小时，2.7小时，下同） 小时

**媒体暴露类型**

1. 最近一周，您平均每天通过传统媒体（如报纸、广播、电视）关注新冠肺炎疫情的时间有多长？ 小时
2. 最近一周，您平均每天通过社交媒体（如微信、QQ、微博）关注新冠肺炎疫情的时间有多长？ 小时
3. 最近一周，您平均每天通过网络新闻（如今日头条、新华网、搜狐新闻）关注新冠肺炎疫情的时间有多长？ 小时
4. 最近一周，您平均每天通过短视频（如抖音、快手）关注新冠肺炎疫情的时间有多长？ 小时

**媒体暴露内容**

1. 最近一周，您每天通过媒体关注最新疫情数据的频率如何？
2. 从不 B. 比较少 C. 中等 D. 比较多 E. 总是
3. 最近一周，您每天通过媒体关注新冠肺炎疫苗研发进展的频率如何？
4. 从不 B. 比较少 C. 中等 D. 比较多 E. 总是
5. 最近一周，您每天通过媒体关注疫情对社会影响的频率如何？
6. 从不 B. 比较少 C. 中等 D. 比较多 E. 总是
7. 最近一周，您每天通过媒体关注疫情对日常生活影响的频率如何？
8. 从不 B. 比较少 C. 中等 D. 比较多 E. 总是
9. 最近一周，您每天通过媒体关注新冠肺炎感染者现状的频率如何？
10. 从不 B. 比较少 C. 中等 D. 比较多 E. 总是
11. 最近一周，您每天通过媒体关注疫情中涌现的英雄事迹的频率如何？
12. 从不 B. 比较少 C. 中等 D. 比较多 E. 总是

**问卷2**

**斯坦福急性应激反应问卷**

下面列表中的内容是人们在经历应激性事件的过程中及事件之后有时会出现的一些体验。请仔细阅读每项题目，根据您过去一周内有关“新冠肺炎疫情”的经历或感受，选择最符合您的选项

| **题目** | **没有体验** | **极少体验** | **偶尔体验** | **有时体验** | **经常体验** | **总是体验** |
| --- | --- | --- | --- | --- | --- | --- |
| 1.我入睡或维持睡眠困难。 | 0 | 1 | 2 | 3 | 4 | 5 |
| 2.我感觉坐立不安。 | 0 | 1 | 2 | 3 | 4 | 5 |
| 3.我有“无时间感的感觉”。 | 0 | 1 | 2 | 3 | 4 | 5 |
| 4.我反应迟缓。 | 0 | 1 | 2 | 3 | 4 | 5 |
| 5.我试图回避与新冠肺炎疫情有关的感受。 | 0 | 1 | 2 | 3 | 4 | 5 |
| 6.我反复做与新冠肺炎疫情有关的噩梦。 | 0 | 1 | 2 | 3 | 4 | 5 |
| 7.如果暴露于使我想起新冠肺炎疫情的情境，我感到异常心烦。 | 0 | 1 | 2 | 3 | 4 | 5 |
| 8.对于小事情我也经常出现惊跳反应。 | 0 | 1 | 2 | 3 | 4 | 5 |
| 9.新冠肺炎疫情使我完成工作或需要做的事情感到困难。 | 0 | 1 | 2 | 3 | 4 | 5 |
| 10.我没有通常存在的我是谁的感觉。 | 0 | 1 | 2 | 3 | 4 | 5 |
| 11.我试图回避使我想起新冠肺炎疫情的活动。 | 0 | 1 | 2 | 3 | 4 | 5 |
| 12.我感觉高度警惕或者“紧张兮兮”。 | 0 | 1 | 2 | 3 | 4 | 5 |
| 13.我感觉自己好像是个陌生人。 | 0 | 1 | 2 | 3 | 4 | 5 |
| 14.我试图回避交谈新冠肺炎疫情。 | 0 | 1 | 2 | 3 | 4 | 5 |
| 15.当暴露于与新冠肺炎疫情有关的提示时，我有身体上的反应。 | 0 | 1 | 2 | 3 | 4 | 5 |
| 16.我回忆新冠肺炎疫情的重要内容有困难。 | 0 | 1 | 2 | 3 | 4 | 5 |
| 17.我试图回避与新冠肺炎疫情有关的想法。 | 0 | 1 | 2 | 3 | 4 | 5 |
| 18.我见到的事物与它们的实际情况感觉有不同。 | 0 | 1 | 2 | 3 | 4 | 5 |
| 19.我反复出现与新冠肺炎疫情有关的不必要的记忆。 | 0 | 1 | 2 | 3 | 4 | 5 |
| 20.我感受不到自己的情感。 | 0 | 1 | 2 | 3 | 4 | 5 |
| 21.我急躁易怒或者发脾气。 | 0 | 1 | 2 | 3 | 4 | 5 |
| 22.我回避与使我想起新冠肺炎疫情的人接触。 | 0 | 1 | 2 | 3 | 4 | 5 |
| 23.我经常突然行动或感受，好像新冠肺炎疫情又再次发生了一样。 | 0 | 1 | 2 | 3 | 4 | 5 |
| 24.我的大脑一片空白。 | 0 | 1 | 2 | 3 | 4 | 5 |
| 25.我忘记了新冠肺炎疫情的大部分过程。 | 0 | 1 | 2 | 3 | 4 | 5 |
| 26.新冠肺炎疫情导致我和其他人的关系出现问题。 | 0 | 1 | 2 | 3 | 4 | 5 |
| 27.我集中注意力困难。 | 0 | 1 | 2 | 3 | 4 | 5 |
| 28.我感觉和其他人疏远或分离。 | 0 | 1 | 2 | 3 | 4 | 5 |
| 29.我感觉新冠肺炎疫情又重新发生了一次的生动体验。 | 0 | 1 | 2 | 3 | 4 | 5 |
| 30.我试图远离使我想起新冠肺炎疫情的地方。 | 0 | 1 | 2 | 3 | 4 | 5 |

**问卷3**

**人口学信息**

1. 您的性别

A.男 B.女

2. 年龄

（ ）岁

3. 婚姻状态

A.单身 B.已婚 C.离异或丧偶

4. 居住地

A.城市 B.城镇 C.农村

**直接暴露程度**

1. 最近一周您是否居住在湖北？

1. 是 B. 否

2. 最近一周您是否有亲朋好友（关系比较亲密人）居住在湖北？

1. 是 B. 否

3. 您身边是否有亲朋好友（关系比较亲密人）被确诊为新冠肺炎？

1. 是 B. 否

**心理疾病史**

您曾经是否有被诊断为患有心理疾病？（例如焦虑症、抑郁症）

1. 没有 B. 一种或多种

**负性生活事件**

最近3年，您是否经历过以下负性生活事件或变故（不包括新冠肺炎疫情）

1. 亲人或朋友死亡

A. 是 B. 否

2. 自己或父母离婚

A. 是 B. 否

3. 自己或家属患重大疾病

A. 是 B. 否

4. 自己或家属遭遇重大交通事故

A. 是 B. 否

5. 遭遇重大自然灾害

A. 是 B. 否

6. 遭遇强烈的家庭冲突

A. 是 B. 否

7. 遭遇刑事处分

A. 是 B. 否

8. 其它重大创伤性事件和变故

A. 是 B. 否

**先前集体创伤暴露**

**汶川大地震暴露**

1. 下面是对汶川大地震的描述，请根据您当时的实际经历进行评估。

A. 没有听说过

B. 只在电视上了解

C. 我居住的地方有轻微震感

D. 我居住的地方有中等程度震感

E. 我居住的地方震感强烈

2. 请根据您的实际情况评估汶川大地震对您造成的创伤程度。

A. 没有创伤 B. 轻度创伤 C. 中等程度创伤 D. 严重创伤 E. 极其严重创伤

**非典疫情（SARS）暴露**

1. 下面是对非典疫情描述，请根据您当时的实际经历进行评估。

A. 没有听说过

B. 只在电视上了解

C. 远离疫情中心

D. 靠近疫情中心

E. 处在疫情中心

2. 请根据您的实际情况评估非典疫情对您造成的创伤程度。

A. 没有创伤 B. 轻度创伤 C. 中等程度创伤 D. 严重创伤 E. 极其严重创伤
